# Opioid-induced adrenal insufficiency: diagnostic and management considerations

**DOI:** 10.3389/fendo.2023.1280603

**Published:** 2024-02-27

**Authors:** Erica Patel, Anat Ben-Shlomo

**Affiliations:** ^1^ Division of Endocrinology, Diabetes & Metabolism, Department of Medicine, Cedars-Sinai Medical Center, Los Angeles, CA, United States; ^2^ Multidisciplinary Adrenal Program, Department of Medicine, Cedars-Sinai Medical Center, Los Angeles, CA, United States

**Keywords:** Adrenal insufficiency, opioids, cortisol, hydrocortisone, narcotics, adrenal insufficiency

## Abstract

The dramatic rise in opioid use over the last two decades has led to a surge in their harmful health effects. Lesser known among clinicians is the impact of opioids on the endocrine system, especially with regard to cortisol. Opioids can suppress the hypothalamus-pituitary-adrenal (HPA) axis and may result in clinically significant adrenal insufficiency, especially in those treated at higher doses and for a longer time. A high clinical suspicion is necessary in this population for early diagnosis of opioid-induced adrenal insufficiency (OAI). Diagnosis of OAI is challenging, as the symptoms are often vague and overlap with those due to opioid use or the underlying pain disorder. Traditional assays to diagnose adrenal insufficiency have not been widely studied in this population, and more investigation is needed to determine how opioids might affect assay results. Once a diagnosis of adrenal insufficiency has been made, glucocorticoid replacement in the form of hydrocortisone is likely the mainstay of treatment, and effort should be made to taper down opioids where possible. Cortisol levels should be retested periodically, with the goal of stopping glucocorticoid replacement once the HPA axis has recovered. In this review, we provide context for diagnostic challenges in OAI, suggest diagnostic tools for this population based on available data, and offer recommendations for the management of this disorder. There is a paucity of literature in this field; given the widespread use of opioids in the general population, more investigation into the effects of opioids on the HPA axis is sorely needed.

## Introduction

The inhibitory effects of opioids on the hypothalamic-pituitary-adrenal (HPA) axis in humans were established years ago (1). However, surprisingly, this fact is frequently underappreciated among clinicians (2). Given the escalating ‘opioid epidemic,’ which has tragically claimed an increasing number of lives over the past two decades (3), there is a pressing need for clinicians to pay closer attention to the suppressive effects of opioids on the HPA axis.

Both acute and chronic opioid treatments have been shown to inhibit adrenocorticotrophic hormone (ACTH) and cortisol production (4, 5), elevating the risk of opioid-induced adrenal insufficiency (OAI). This effect might occur through a secondary mechanism, possibly involving inhibition of corticotrophin-releasing hormone (CRH) action on pituitary corticotrophs (6). Although not every reduction in cortisol is clinically relevant and warrants full workup for OAI, a high index of suspicion is prudent for patients treated with high-dose opioids and/or for prolonged periods (7). It is worth noting that prolonged opioid treatment was not shown to inhibit aldosterone in humans (5).

Data on the prevalence of OAI are primarily derived from small, retrospective studies that lack uniformity in setting, opioid dose, duration, or route of administration (**Table 1**). Additionally, not all studies employ rigorous or updated diagnostic testing for adrenal insufficiency. Clinical practice guidelines for management of hypopituitarism recommend screening for adrenal insufficiency with morning cortisol [AI is more likely if <3 mcg/dL (100 nmol/L)], and ACTH^1-24^ (250 mcg) stimulation testing for confirmation. Peak cortisol ≤18 mcg/dL (500 nmol/L) at 30 or 60 minutes after ACTH^1-24^ stimulation is diagnostic for adrenal insufficiency according to older cortisol assays (13). However, according to recent study, a lower threshold of <14-15 mcg/dL (386.3-413.9 nmol/L) at 30 minutes post stimulation should be used for validation of adrenal insufficiency (14). Only one study utilized a lower cortisol cutoff of 14.7 mcg/dL (405 nmol/L) measured at 25-70 minutes after ACTH^1-24^ stimulation in a retrospective evaluation demonstrating OAI in 4% of 75 patients on chronic opioid treatment (11). Moreover, diagnostic criteria or OAI are not uniformly applied across studies. In a systematic review and meta-analysis evaluating endocrine effects of opioids, only 8 of 21 studies assessing the effects on the HPA axis used stimulation testing to measure cortisol, and these studies used insulin-induced hypoglycemia and ACTH^1-24^ stimulation testing using the older cortisol cutoffs or other assays such as CRH, metyrapone, and yohimbine. They calculated prevalence of OAI to be 5-42% with weighted mean percentage of 15% (6). In a prospective cross section study of 102 patients receiving opioids and referred to a pain rehabilitation clinic, 9% (n=9) were diagnosed with OAI, but criteria included clinical presentation, morning cortisol ≤10 mcg/dL (277 nmol/L), ACTH ≤15 pg/ml (3.3 pmol/L), and dehydroepiandrosterone sulfate (DHEAS) ≤25 mcg/dL (86.7 nmol/L), as well as peak cortisol level of 16-17 mcg/dL (441.4-469 nmol/L) during an ACTH^1-24^ stimulation test in 3 patients (8), which is higher than the new cortisol cutoff suggested. Given the high rates of opioid use in the community, the number of patients at risk for OAI and its associated morbidity and mortality is substantial.

**Table 1:** Prevalence of OAI Among Patients Receiving Chronic Opioid Therapy.

	N	Inclusion Criteria*	MME dose, median mg (range)	Diagnostic Criteria	Prevalence of OAI
Cross-Sectional Studies					
Li, 2020 (8)	102	Intermittent or continuous opioid use for the last 90 days	60 (3-840)	Serum ACTH <16 pg/mL (3.5 pmol/L), morning cortisol < 11 mcg/dL (303.5 nmol/L), DHEAS < 26 mcg/dL (90.1 nmol/L), DHEAS <26 mcg/dL, ACTH^1-24^ stimulation test with peak cortisol < 18 mcg/dL (496.6 nmol/L)	9% overall; 6% based on cortisol, serum ACTH, and DHEAS, 3% also based on stimulation test
Lamprecht, 2018 (9)	40	Opioid use >6 months, ≥25 mg MEDD	74 (25–265)	ACTH^1-24^ stimulation test with peak cortisol cortisol < 18 mcg/dL (496.6 nmol/L), overnight metyrapone stimulation test (30 mg/kg)	cortisol < 18 mcg/dL (496.6 nmol/L)
Gibb, 2016 (10)	48	Opioid use >6 months	68 (40-153)	ACTH^1-24^ stimulation test with peak cortisol <15.6 mcg/dL (430 nmol/L), 8 AM cortisol <3.62 mcg/dL (100 nmol)	6.25% based on stimulation test, 8.3% with low morning cortisol
Retrospective Studies					
Colling, 2022 (11)	75	Opioid use >30 days	83 (23-193)	ACTH^1-24^ stimulation test with two different peak cortisol cutoffs: 1. with peak cortisol <14.7 mcg/dL (405 nmol/L) 2. <18.1 mcg/dL (500 nmol/L)	4% with <14.7 mcg/dL (405 nmol/L) 18.7% with <18.1 mcg/dL (500 nmol/L)
Merdin, 2016 (12)	20	≥25 mg MEDD for ≥1 month for cancer pain and VAS <2	180 (10-420)	Serum cortisol < 4.3 mcg/dL (118.6 nmol/L)	cortisol < 4.3 mcg/dL (118.6 nmol/L)
Abs, 2000 (5)	73	Intrathecal opioid therapy	4.8 (0.6-15)	24h UFC <20 mg/L, ITT <180 µg/L	19.7% based on 24h UFC, < 20 mcg/dL (551.8 nmol/L) ITT peak cortisol < 18 [not 180] mcg/dL [not mcg/L] (496.6 nmol/L)

*Patients with noncancer pain unless otherwise noted.

ACTH, adrenocorticotrophic hormone; DHEAS, dehydroepiandrosterone sulfate; ITT, insulin tolerance test; MEDD, morphine equivalent daily dose; MME, morphine milligram equivalent; OAI, opioid-induced adrenal insufficiency; UFC, urine [not urinary] free cortisol ; VAS, visual analog scale.

This article suggests best practices for diagnosis and management of OAI in current clinical practice and identifies key areas in which further study of this often-overlooked disorder can improve patient outcomes.

## Diagnostic considerations in OAI

Signs and symptoms of OAI are nonspecific, and include fatigue, lack of appetite, weight loss, gastrointestinal symptoms such as nausea, vomiting, diarrhea, mood changes, signs such as orthostatic hypotension, biochemical findings such as hyponatremia, and impaired quality of life; moreover, they may overlap with symptoms of opioid treatment and the disorders causing pain for which opioids are prescribed (11).

Once OAI is clinically suspected, the question of which assay to use remains. The cutoffs recommended by the Endocrine Society guidelines (13) for confirmation of adrenal insufficiency from other causes, i.e., screening morning cortisol <3 mcg/dL (100 nmol/L) and baseline cortisol ≥18 mcg/dL (500 nmol) at 30 and 60 minutes after ACTH^1-24^ stimulation, have not been prospectively validated in opioid-treated patients. The refined cutoff for newer immunoassays and mass spectrometry, i.e., 14-15 mcg/dL (386-414 nmol/L) 30 minutes after stimulation (14), was retrospectively assessed in OAI (11) but prospective validation has not been conducted. This is a crucial issue, as the cutoffs established for central adrenal insufficiency might not be applicable to OAI. For example, morning total cortisol threshold levels were established in outpatients without specific consideration of pain, yet pain, as with all other stressogenic conditions, stimulates the HPA axis (15), skewing accurate assessment of cortisol level independent of any effect from the opioids. In addition, both pain and opioids have been shown to alter the circadian rhythm that controls the HPA axis (16), which, in turn, suggests the need for a different cutoff for morning cortisol screening tests particularly among hospitalized patients (11).

Even if it were possible to control for the effects of pain and opioid use on tests that rely on total cortisol levels, multiple other factors that may co-exist in opioid-treated patients might affect their reliability in confirming a diagnosis of secondary adrenal insufficiency regardless of the etiology (17). The limited sensitivity and specificity of these tests have been described in patients with conditions affecting total circulating protein (albumin and corticosteroid binding globulin), including kidney and liver diseases and critical illnesses (18), and a meta-analysis of studies evaluating diagnostic performance of the ACTH^1-24^ stimulation test for secondary adrenal insufficiency shows high specificity but low sensitivity even in unselected patients (19).

Considering the known challenges with current diagnostic assessments for adrenal insufficiency, and given the lack of optimal validation in patients with suspected OAI, we suggest the following diagnostic approach for patients treated with long-term, high-dose opioids. These patients should be periodically assessed for new symptoms suggestive of OAI, such as fatigue, reduced appetite and weight loss, gastrointestinal disturbance, mood changes, hypotension, and hyponatremia, while also recognizing that similar adverse effects may be due to opioid use itself or the disease that causes the pain for which they receive treatment with opioids (20). If the patient manifests symptoms of adrenal insufficiency, we suggest the clinician refer the patient to an endocrinologist for further evaluation. The endocrinologist should carefully review the patient’s medical history to address the potential contribution of any other medications that might affect total cortisol levels, including any glucocorticoid formula (glucocorticoid injections are often used for the treatment of pain); antifungals or other steroidogenesis inhibitors; somatostatin analogues; or drugs that change the activity of CYP3A4, an enzyme responsible for the metabolism of cortisol. All other possible etiologies for adrenal insufficiency resulting from infections, bilateral bleeding, tumors, surgery and autoimmune disorders should be considered as well.

Patients with intense pain often cannot stop taking opioid medications, which would allow the HPA axis to recover from an acute opioid suppression and for morning cortisol to reach the true endogenous levels. Therefore, screening with morning cortisol is less likely to be informative in cases of suspected OAI. Rather, we suggest the pharmacological high-dose (250 mcg) ACTH^1-24^ stimulation test be administered in all suspicious cases. Importantly, given the rapid effect of acute opioid administration on the HPA axis, it is reasonable to avoid administration of opioids for several hours prior to testing and for as long as tolerated by the patient in an attempt to allow for the true endogenous cortisol levels to manifest. For further confirmation, we also suggest measuring cortisol at the two established time points of 30 and 60 minutes after stimulation. Failure to exceed a threshold of 14-15 mcg/dL (386-414 nmol/L) at 30 minutes and/or failure to exceed the threshold of 18 mcg/dL (496.6 nmol/L) at 60 minutes in the setting of long-term, high-dose opioid use supports a diagnosis of OAI. Baseline clinical evaluation and measurement of cortisol levels can be considered before initiating opioid treatment to better assess patients during follow up for the development of OAI.

The combination of morning cortisol, low-normal to low ACTH, and low DHEAS has been proposed as a possible diagnostic marker for OAI (8), but its role might be limited. The challenges of relying on morning cortisol in screening for OAI were discussed above. Measurement of ACTH is not utilized in the initial diagnosis of adrenal insufficiency, but can be an indicator of where along the HPA axis the deficiency is located (21). Low ACTH in the presence of a failure to pass the ACTH^1-24^ stimulation test is indicative of secondary adrenal insufficiency, which supports the diagnosis of OAI due to a central cause; however, opioid receptors are abundant on the adrenal glands as well (22) and the presence of a combined primary (which will cause high ACTH level) and secondary (causing low ACTH level) adrenal insufficiency resulting in low-normal ACTH level had never been studied. Nevertheless, if results of the ACTH^1-24^ stimulation test support a diagnosis of adrenal insufficiency, ACTH levels should be measured to distinguish between primary adrenal insufficiency at the level of the adrenal glands or central adrenal insufficiency at the level of the pituitary or the hypothalamus (21). DHEAS largely originates in the adrenal gland, has a long half-life of approximately 24 hours, and is regulated by ACTH (23). Accordingly, it may reflect ACTH deficiency and has been studied as a potential additive biomarker to ACTH^1-24^ stimulation in the diagnosis of adrenal insufficiency (24, 25). However, DHEAS levels decrease with age (26) and with chronic diseases such as diabetes mellitus (27), autoimmune disease (28), and cancer (29), and the lack of OAI-specific, age-specific reference ranges all may limit its use. Nevertheless, as access to an endocrinologist and ACTH^1-24^ stimulation testing may not be readily available to all clinicians, the combination of low/low-normal morning cortisol, DHEAS, and ACTH levels several hours after the last dose of opioids may be used as a preliminary screening tool for OAI.

## Management considerations in OAI

After establishing a diagnosis of OAI, treatment decision-making remains challenging, as there are no large, prospective, randomized, placebo-controlled clinical trials of patients diagnosed according to accepted standards to offer guidance on optimal approaches and outcomes.

To date, the only prospectively designed study included 17 patients treated with mean morphine milligram equivalent (MME) dose of ≥20 mg/day for at least 4 weeks for noncancer pain who were diagnosed with hypocortisolism based on plasma cortisol levels ≤12.7 mcg/dL (≤350 nmol/L) using the cold pressure test (CPT), in which exposure to cold stimulates nociceptive neuronal pathways that activate the HPA axis (30). Patients received hydrocortisone replacement (10 mg/m^2^/day given in 3 daily divided doses) or placebo for 28 days, then switched to the opposite treatment after 10-day washout. Health status questionnaires completed before and after each 28-day treatment period showed that treatment with hydrocortisone resulted in better outcomes on the SF-36 bodily pain and vitality domains, as well as greater improvements in general activity, mood, and work on the Pain Interference Score and on pain response threshold and tolerance on the CPT. No improvement was noted on the AddiQoL survey specific to symptoms of adrenal insufficiency, or on the Brief Pain Inventory-Short Form survey or the Pain Severity Score survey (30). Although these data suggest that hormone replacement in OAI could positively impact overall patient outcomes, the CPT is not used in standard endocrinology practice to diagnose adrenal insufficiency and the 12.7 mcg/dL (350.4 nmol/L) cortisol threshold has never been validated using the gold-standard insulin tolerance test or the ACTH^1-24^ stimulation test for this purpose.

Nevertheless, results of a larger retrospective study in 40 patients with more rigorously defined OAI (peak cortisol level ≤18 mcg/dL [497 nmol/L] after 250 mcg ACTH^1-24^ stimulation) who were treated with higher doses of opioid (median MME dose 105 mg/day [range, 60-200 mg/day]) for a longer period of time (median duration 60 months [range, 3-360 months]) also suggests clinical benefit with glucocorticoid replacement (31). Comparing symptoms reported before and after treatment, fatigue improved in 48% of patients, weight loss in 41%, abdominal pain and nausea in 25%, and headache in 17%. Notably, 38% ultimately tapered and/or stopped opioids, and HPA axis recovery was evident in 70% of these patients on biochemical follow up (31). Reports of individual patients with OAI treated with hydrocortisone describe similar benefits (22).

Considering the scarce data available on OAI treatment, we suggest a treatment approach that aims to replace the deficient endogenous cortisol and provide symptomatic relief. Thus, in cases where OAI is strongly suspected based on clinical and biochemical presentation, treatment with hydrocortisone should be considered following the Endocrine Society guidelines of 15-20 mg per day, divided into 2-3 doses, with the highest dose taken in the morning (13). As long-term treatment with opioids does not adversely affect plasma renin activity and aldosterone production (32), mineralocorticoid replacement is likely not required in patients with OAI.

Lastly, there is no protocol to suggest how to follow hydrocortisone-treated OAI patients. Case reports suggest cessation of opioids or even reduction of opioids doses can lead to reversal of OAI (33) (**Table 2** **).** However, the lowest opioid dose at which the HPA axis recovers and the time to recovery are both unknown. Therefore, per clinician discretion, periodic clinical and biochemical assessment of OAI symptoms during continuous treatment and dose tapering is suggested.

**Table 2:** Efficacy of Glucocorticoid Treatment in Patients With OAI.

	Study Type	N	Opioid Use*	OIA Assessment	Intervention	Outcomes	Follow-up
Nenke, 2015 (30)	Randomized, double-blind, placebo-controlled crossover	17	MEDD ≥20 mg for ≥4 weeks (47% 20-50 mg, 53% >100 mg)	Plasma cortisol ≤12.7 mcg/dL (350 nmol/L) after cold pressor test	28 days of 10 mg/m^2^/d oral hydrocortisone TID or placebo, 2-week washout, then crossover for 28 days	Improvement in bodily pain and vitality on SF-36; improvement on general activity, mood, and work on Pain Interference Score	Not reported
Li, 2020 (31)	Retrospective cohort	40	Median MME 105 mg/d (range, 60-200) for median 96 months (range, 24-120)	Low AM cortisol, DHEAS, and/or ACTH (59%); peak cortisol <= 18 mcg/dL (496.6 nmol/L) after ACTH^1-24^ stimulation (41%)	Hydrocortisone (95%) or prednisone (5%); dose not reported	Improvement in fatigue, musculoskeletal pain, and weight loss	Symptomatic improvement in 70% of patients with clinical follow-up, resolution of OAI in 70% of patients with biochemical follow-up
Gibb, 2016 (10)	Prospective, cross-sectional; reports on patients who did not pass ACTH^1-24^ stimulation test	2	39-year-old female on 185 mg/d MME 37-year-old female on 100 mg/d MME	Peak cortisol <437 nmol/L (15.9 mcg/dL) after ACTH^1-24^ stimulation	Hydrocortisone 10 mg AM/5 mg PM	Improvement in lethargy and fatigue	Not reported
Kondo, 2022 (34)	Case report	1	46-year-old female on 90-120 mg/d MME for 48 months (transdermal fentanyl)	60-minute cortisol 15 mcg/dL (413.8 nmol/L) after ACTH^1-24^ stimulation; AM ACTH 4.7 pg/ml (1 pmol/L), AM cortisol 2.1 mcg/dL (57.9 nmol/L), DHEAS 39 mcg/dL (135.2 nmol/L)	Hydrocortisone 15 mg/d	Partial improvement in fatigue, appetite, and vitality	Unable to taper off opioid; hydrocortisone continued
Debono 2011 (35)	Case report	1	21-year-old female on 100 mg tramadol 4x/d	30-min cortisol 307 nmol/l and 60-min cortisol 419 nmol/l after ACTH^1-24^ stimulation; AM ACTH 9.7 ng/L			

*Patients with noncancer pain unless otherwise noted.

MEDD, morphine equivalent daily dose; MME, morphine milligram equivalent; OAI, opioid-induced adrenal insufficiency.

## Conclusions

Opioid treatment is associated with the development of OAI in a small subset of patients, and those treated for a longer time and at higher doses might be at increased risk. Active monitoring for OAI in these patients is warranted. As no specific diagnostic criteria are available for OAI, at this time, treating clinician should rely on Endocrine Society guidelines for diagnosis of central adrenal insufficiency. It is preferable that patients be diagnosed, treated, and monitored by a skilled endocrinologist. Patients should be periodically monitored for new clinical symptoms and signs of adrenal insufficiency and, if suspicious, ACTH^1-24^ stimulation testing should be considered to confirm the diagnosis. If stimulation testing is unavailable, a combination of morning cortisol, DHEAS, and ACTH measurement may be used as initial screening. All tests should be performed several hours after the last opioid dose to avoid the acute HPA axis suppression observed shortly after opioid treatment.

If both clinical and biochemical evaluation suggest a high probability of OAI, the clinician should consider treatment with daily hydrocortisone as recommended for patients with secondary adrenal insufficiency from other causes. Treatment should be temporary in most cases while continuously monitoring HPA axis recovery, and hydrocortisone dose should be tapered down as opioid dose is reduced and HPA axis function recovers.

## Author contributions

EP: Data curation, Writing – original draft. AB-S: Data curation, Writing – original draft, Conceptualization, Formal analysis, Writing – review & editing.
